# *rac*-*N*,*N*′-Dimethyl-*N*,*N*′-(1,1′-binaphthyl-2,2′-di­yl)diformamide

**DOI:** 10.1107/S160053681104640X

**Published:** 2011-11-09

**Authors:** Jing Zeng, Seik Weng Ng

**Affiliations:** a College of Chemistry, Xinjiang Normal University, Urumqi 830054, People’s Republic of China; b Department of Chemistry, University of Malaya, 50603 Kuala Lumpur, Malaysia; c Chemistry Department, Faculty of Science, King Abdulaziz University, PO Box 80203 Jeddah, Saudi Arabia

## Abstract

The mol­ecule of the title compound, C_24_H_20_N_2_O_2_, lies on a twofold rotation axis that relates one 2-(*N*-methyl­formamido)­naphthyl unit to the other. The *N*-methyl­formamido substituent is twisted by 54.9 (1)° with respect to the naphthalene fused-ring system; the two fused-ring systems are themselves twisted by 70.3 (1)°.

## Related literature

For the synthesis of 2,2′-bis­(methyl­amino)-1,1′-binaphthyl, see: Miyano *et al.* (1984 ▶).
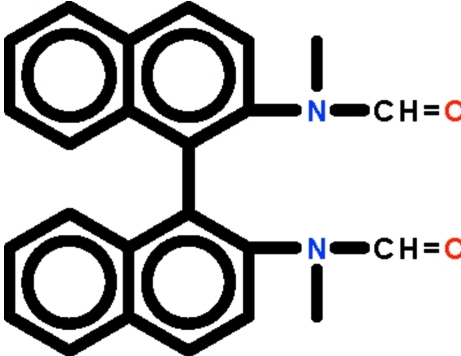


## Experimental

### 

#### Crystal data

C_24_H_20_N_2_O_2_
*M* *_r_* = 368.42
Tetragonal, 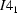

*a* = 11.6548 (12) Å
*c* = 13.9171 (15) Å
*V* = 1890.4 (3) Å^3^
*Z* = 4
Mo *K*α radiation
μ = 0.08 mm^−1^
*T* = 113 K
0.34 × 0.20 × 0.16 mm

#### Data collection

Rigaku Saturn CCD area-detector diffractometer
Absorption correction: multi-scan (*CrystalClear*; Rigaku/MSC, 2005 ▶) *T*_min_ = 0.972, *T*_max_ = 0.987
11169 measured reflections
1091 independent reflections
1051 reflections with *I* > 2σ(*I*)
*R*_int_ = 0.044

#### Refinement

*R*[*F* ^2^ > 2σ(*F* ^2^)] = 0.038
*wR*(*F* ^2^) = 0.101
*S* = 1.07
1091 reflections
129 parameters
1 restraint
H-atom parameters constrained
Δρ_max_ = 0.20 e Å^−3^
Δρ_min_ = −0.14 e Å^−3^

### 

Data collection: *CrystalClear* (Rigaku/MSC, 2005 ▶); cell refinement: *CrystalClear*; data reduction: *CrystalClear*; program(s) used to solve structure: *SHELXS97* (Sheldrick, 2008 ▶); program(s) used to refine structure: *SHELXL97* (Sheldrick, 2008 ▶); molecular graphics: *X-SEED* (Barbour, 2001 ▶); software used to prepare material for publication: *publCIF* (Westrip, 2010 ▶).

## Supplementary Material

Crystal structure: contains datablock(s) global, I. DOI: 10.1107/S160053681104640X/xu5377sup1.cif

Structure factors: contains datablock(s) I. DOI: 10.1107/S160053681104640X/xu5377Isup2.hkl

Supplementary material file. DOI: 10.1107/S160053681104640X/xu5377Isup3.cml

Additional supplementary materials:  crystallographic information; 3D view; checkCIF report

## Figures and Tables

**Fig. 1.**
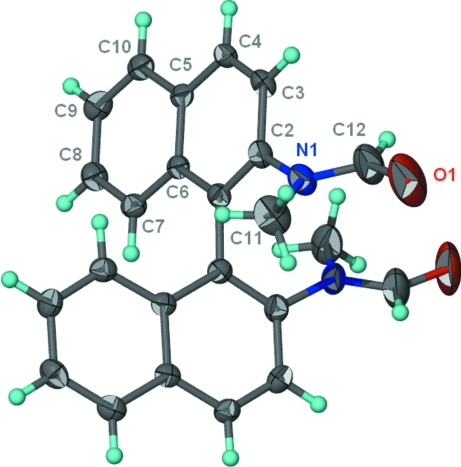
Thermal ellipsoid plot (Barbour, 2001) of C24H20N2O2 at the 70% probability level; hydrogen atoms are drawn as spheres of arbitrary radius. The unlabeled atoms are related to the labeled one by 2 - x, -y, z.

## supplementary crystallographic information

### Comment

We report here the methylation of commercially available *racemic* 2,2'-diamino-1,1'-binaphthyl to yield 2,2'-bis(methylamino)-1,1'-binaphthyl. The compound should react with triethyl orthoformate to yield an imidzolium salt. The reaction with ethyl formate, an unintended similar-sounding reagent, gave 2,2'-bis(*N*-methyformido)-1,1'-binaphthyl (Scheme I) instead.

The C_24_H_20_N_2_O_2_ molecules lies on a twofold rotation axis that relates one *N*-methylformamido)-1-naphthyl moiety to the other (Fig. 1). The *N*-methylformamido substituent is twisted by 54.9 (1) ° with respect to the naphathalene fused-ring; the two fused-rings are themselves twisted by 70.3 (1) °.

### Experimental

2,2'-Bis(methylamino)-1,1'-binaphthyl was prepared by treatment of *racemic* 2,2'-diamino-1,1'-binaphthyl (purchased from Aldrich Chemical Company) with ethyl chloroformate in benzene in the presence of pyridine, followed by reduction with lithium aluminium hydride in tetrahydrofuran (Miyano *et al.*, 1984). An ethyl formate solution (10 ml) of 2,2'-bis(methylamino)-1,1'-binaphthyl (156 mg, 0.50 mmol) was heated at 327 K for 10 h. The solvent was removed under reduced pressure and the residue was purified by column chromatography (eluent: ethyl acetate/petroleum ether 10/1) to give the title compound (158 mg, 86%) as a white solid. Crystals were obtained upon recrystallized from a dichloromethane-hexane mixture.

### Refinement

Carbon-bound H-atoms were placed in calculated positions (C–H 0.95–0.98 Å) and were included in the refinement in the riding model approximation, with *U*_iso_(H) set to 1.2–1.5*U*_eq_(C). In the absence of heavy atoms, 1000 Friedel pairs were merged.

### Crystal data

**Table:**

C_24_H_20_N_2_O_2_	*D*_x_ = 1.294 Mg m^−^^3^
*M**_r_* = 368.42	Mo *K*α radiation, λ = 0.71070 Å
Tetragonal, *I*4_1_	Cell parameters from 2958 reflections
Hall symbol: I 4bw	θ = 2.3–27.1°
*a* = 11.6548 (12) Å	µ = 0.08 mm^−^^1^
*c* = 13.9171 (15) Å	*T* = 113 K
*V* = 1890.4 (3) Å^3^	Prism, colorless
*Z* = 4	0.34 × 0.20 × 0.16 mm
*F*(000) = 776	

### Data collection

**Table:**

Rigaku Saturn CCD area-detector diffractometer	1091 independent reflections
Radiation source: rotating anode	1051 reflections with *I* > 2σ(*I*)
confocal	*R*_int_ = 0.044
Detector resolution: 7.31 pixels mm^-1^	θ_max_ = 27.1°, θ_min_ = 2.3°
ω and φ scans	*h* = −14→14
Absorption correction: multi-scan (*CrystalClear*; Rigaku/MSC, 2005)	*k* = −14→14
*T*_min_ = 0.972, *T*_max_ = 0.987	*l* = −17→17
11169 measured reflections	

### Refinement

**Table:**

Refinement on *F*^2^	Secondary atom site location: difference Fourier map
Least-squares matrix: full	Hydrogen site location: inferred from neighbouring sites
*R*[*F*^2^ > 2σ(*F*^2^)] = 0.038	H-atom parameters constrained
*wR*(*F*^2^) = 0.101	*w* = 1/[σ^2^(*F*_o_^2^) + (0.0702*P*)^2^ + 0.3273*P*] where *P* = (*F*_o_^2^ + 2*F*_c_^2^)/3
*S* = 1.07	(Δ/σ)_max_ = 0.001
1091 reflections	Δρ_max_ = 0.20 e Å^−^^3^
129 parameters	Δρ_min_ = −0.14 e Å^−^^3^
1 restraint	Extinction correction: *SHELXL97* (Sheldrick, 2008), Fc^*^=kFc[1+0.001xFc^2^λ^3^/sin(2θ)]^-1/4^
Primary atom site location: structure-invariant direct methods	Extinction coefficient: 0.012 (3)

### Fractional atomic coordinates and isotropic or equivalent isotropic displacement parameters (Å^2^)

**Table:**

	*x*	*y*	*z*	*U*_iso_*/*U*_eq_	
O1	0.8311 (3)	−0.0321 (2)	0.33342 (16)	0.0772 (9)	
N1	0.85923 (17)	0.00005 (15)	0.17381 (13)	0.0284 (5)	
C1	0.96952 (16)	0.05699 (16)	0.03106 (14)	0.0187 (4)	
C2	0.89065 (18)	0.08195 (17)	0.10211 (15)	0.0215 (4)	
C3	0.83669 (17)	0.19135 (17)	0.10642 (15)	0.0220 (4)	
H3	0.7816	0.2067	0.1551	0.026*	
C4	0.86360 (17)	0.27432 (16)	0.04105 (15)	0.0199 (4)	
H4	0.8286	0.3477	0.0459	0.024*	
C5	0.94283 (16)	0.25287 (15)	−0.03388 (14)	0.0176 (4)	
C6	0.99650 (16)	0.14242 (15)	−0.03944 (14)	0.0172 (4)	
C7	1.07543 (16)	0.12335 (17)	−0.11571 (15)	0.0204 (4)	
H7	1.1138	0.0516	−0.1202	0.024*	
C8	1.09713 (17)	0.20650 (18)	−0.18289 (15)	0.0229 (4)	
H8	1.1490	0.1911	−0.2339	0.028*	
C9	1.04310 (17)	0.31514 (18)	−0.17716 (16)	0.0241 (5)	
H9	1.0591	0.3724	−0.2239	0.029*	
C10	0.96813 (17)	0.33714 (17)	−0.10433 (16)	0.0222 (4)	
H10	0.9322	0.4102	−0.1007	0.027*	
C11	0.8095 (2)	−0.11084 (19)	0.1472 (2)	0.0391 (6)	
H11A	0.8619	−0.1726	0.1663	0.059*	
H11B	0.7976	−0.1134	0.0775	0.059*	
H11C	0.7358	−0.1208	0.1800	0.059*	
C12	0.8630 (3)	0.0295 (2)	0.26773 (19)	0.0475 (8)	
H12	0.8926	0.1030	0.2836	0.057*	

### Atomic displacement parameters (Å^2^)

**Table:**

	*U*^11^	*U*^22^	*U*^33^	*U*^12^	*U*^13^	*U*^23^
O1	0.149 (3)	0.0543 (14)	0.0279 (12)	0.0017 (15)	0.0288 (14)	0.0093 (10)
N1	0.0414 (11)	0.0224 (9)	0.0214 (10)	−0.0013 (8)	0.0095 (8)	0.0025 (7)
C1	0.0213 (9)	0.0166 (9)	0.0182 (9)	−0.0009 (7)	−0.0013 (7)	−0.0022 (7)
C2	0.0268 (10)	0.0200 (9)	0.0179 (9)	−0.0023 (8)	0.0009 (8)	−0.0008 (8)
C3	0.0232 (10)	0.0226 (10)	0.0203 (9)	−0.0009 (7)	0.0022 (8)	−0.0053 (8)
C4	0.0229 (9)	0.0173 (9)	0.0196 (10)	0.0014 (7)	−0.0029 (8)	−0.0049 (7)
C5	0.0174 (9)	0.0166 (9)	0.0190 (9)	−0.0011 (7)	−0.0042 (7)	−0.0016 (8)
C6	0.0172 (9)	0.0163 (9)	0.0179 (9)	−0.0010 (7)	−0.0035 (7)	−0.0020 (7)
C7	0.0197 (9)	0.0195 (9)	0.0220 (10)	0.0011 (7)	−0.0021 (8)	−0.0013 (8)
C8	0.0219 (9)	0.0255 (10)	0.0215 (10)	−0.0019 (8)	0.0035 (8)	−0.0003 (8)
C9	0.0253 (10)	0.0211 (10)	0.0259 (11)	−0.0051 (8)	−0.0012 (8)	0.0058 (8)
C10	0.0231 (10)	0.0188 (10)	0.0246 (10)	−0.0011 (7)	−0.0052 (8)	−0.0012 (8)
C11	0.0523 (15)	0.0288 (12)	0.0361 (13)	−0.0120 (10)	0.0130 (12)	0.0041 (10)
C12	0.084 (2)	0.0345 (14)	0.0237 (12)	0.0020 (13)	0.0099 (13)	0.0022 (10)

### Geometric parameters (Å, °)

**Table:**

O1—C12	1.220 (4)	C5—C6	1.433 (2)
N1—C12	1.352 (3)	C6—C7	1.422 (3)
N1—C2	1.429 (3)	C7—C8	1.370 (3)
N1—C11	1.464 (3)	C7—H7	0.9500
C1—C2	1.381 (3)	C8—C9	1.416 (3)
C1—C6	1.433 (3)	C8—H8	0.9500
C1—C1^i^	1.507 (4)	C9—C10	1.363 (3)
C2—C3	1.423 (3)	C9—H9	0.9500
C3—C4	1.364 (3)	C10—H10	0.9500
C3—H3	0.9500	C11—H11A	0.9800
C4—C5	1.415 (3)	C11—H11B	0.9800
C4—H4	0.9500	C11—H11C	0.9800
C5—C10	1.419 (3)	C12—H12	0.9500
			
C12—N1—C2	119.83 (19)	C8—C7—C6	121.21 (18)
C12—N1—C11	118.8 (2)	C8—C7—H7	119.4
C2—N1—C11	120.98 (19)	C6—C7—H7	119.4
C2—C1—C6	119.34 (16)	C7—C8—C9	120.79 (19)
C2—C1—C1^i^	119.98 (15)	C7—C8—H8	119.6
C6—C1—C1^i^	120.61 (15)	C9—C8—H8	119.6
C1—C2—C3	120.87 (18)	C10—C9—C8	119.68 (19)
C1—C2—N1	122.00 (17)	C10—C9—H9	120.2
C3—C2—N1	117.12 (18)	C8—C9—H9	120.2
C4—C3—C2	120.35 (18)	C9—C10—C5	121.14 (18)
C4—C3—H3	119.8	C9—C10—H10	119.4
C2—C3—H3	119.8	C5—C10—H10	119.4
C3—C4—C5	121.08 (17)	N1—C11—H11A	109.5
C3—C4—H4	119.5	N1—C11—H11B	109.5
C5—C4—H4	119.5	H11A—C11—H11B	109.5
C4—C5—C10	121.50 (16)	N1—C11—H11C	109.5
C4—C5—C6	118.90 (17)	H11A—C11—H11C	109.5
C10—C5—C6	119.59 (18)	H11B—C11—H11C	109.5
C7—C6—C5	117.58 (17)	O1—C12—N1	124.4 (3)
C7—C6—C1	122.99 (16)	O1—C12—H12	117.8
C5—C6—C1	119.42 (17)	N1—C12—H12	117.8

Symmetry codes: (i) −*x*+2, −*y*, *z*.
